# Characterization and Anti-Inflammatory Effects of *Akkermansia muciniphila*-Derived Extracellular Vesicles

**DOI:** 10.3390/microorganisms13020464

**Published:** 2025-02-19

**Authors:** Sasa Zhao, Jie Xiang, Minhazul Abedin, Jingyi Wang, Zhiwen Zhang, Zhongwei Zhang, Hua Wu, Junsong Xiao

**Affiliations:** 1 School of Food and Health, Beijing Technology and Business University, 33 Fucheng Road, Haidian District, Beijing 100048, China; zhaosasa2022@163.com (S.Z.); xiangjie0701@126.com (J.X.); wjy08280729@163.com (J.W.); zhangzhiwen0604@163.com (Z.Z.); 2 School of Light Industry Science ang Engineering, Beijing Technology and Business University, 33 Fucheng Road, Haidian District, Beijing 100048, China; abedin92@gmail.com (M.A.); zhangzhongwei113@gmail.com (Z.Z.)

**Keywords:** *A. muciniphilia*, Am-EVs, Caco-2, inflammatory, MAPK

## Abstract

Bacterial extracellular vesicles (EVs) play a pivotal role in host–microbe communication. *Akkermansia muciniphila*, a symbiotic bacterium essential for intestinal health, is hypothesized to exert its effects via EVs. Here, we successfully isolated and characterized EVs derived from *A. muciniphila* (Am-EVs) using ultracentrifugation. Am-EVs exhibited a double-membrane structure, with an average diameter of 92.48 ± 0.28 nm and a proteomic profile comprising 850 proteins. In an in vitro model of lipopolysaccharide (LPS)-induced inflammation in human colorectal adenocarcinoma cells (Caco-2), treatment with both 25 and 50 μg/mL Am-EVs significantly reduced oxidative stress markers, including reactive oxygen species (ROS), nitric oxide (NO), and malondialdehyde (MDA), while restoring catalase activity (CAT). Am-EVs also suppressed the expression of pro-inflammatory cytokines tumor necrosis factor alpha (TNF-α), interleukin-1 beta (IL-1β), and interleukin-6 (IL-6). Subsequent transcriptomic sequencing and Western blot experiments revealed that Am-EVs attenuate the MAPK signaling pathway by downregulating TRIF, MyD88, p38 MAPK, and FOS while upregulating TGFBR2. These findings suggest that Am-EVs mediate anti-inflammatory effects through modulation of MAPK signaling, highlighting their potential as therapeutic agents in intestinal inflammation.

## 1. Introduction

*Akkermansia muciniphila*, classified within the phylum Verrucomicrobiae, is a Gram-negative anaerobic bacterium that relies exclusively on host-derived mucin glycoproteins as its sources of carbon and nitrogen for growth [1]. In the context of healthy gut microbiota, this bacterium constitutes approximately 3–5% of the microbial population [2]. *A. muciniphila* is a distinctive intestinal commensal organism that plays a crucial role in regulating intestinal barrier function, maintaining homeostasis, and participating in metabolic processes [3]. Numerous studies have elucidated the robust association between *A. muciniphila* and gut-related metabolic disorders. For example, the abundance of *A. muciniphila* is markedly reduced in the intestines of individuals with inflammatory bowel disease, colorectal cancer, obesity, and diabetes mellitus. However, its levels significantly increase following treatment with medications that potentially ameliorate these conditions and restore intestinal tract integrity [4,5,6,7]. Subsequent research on animals has demonstrated that introducing more *A. muciniphila* into mice with an enteritis model can help restore the intestinal barrier and improve disease-related indicators [8]. This suggests that *A. muciniphila* plays a role in maintaining the homeostasis of the intestinal barrier.

Bacterial extracellular vesicles (EVs) are nanosized spherical structures with a lipid bilayer, ranging in diameter from 20 to 300 nm, secreted by bacteria [9]. These vesicles, which encapsulate a range of active ingredients, such as lipids, proteins, peptidoglycans, and nucleic acids, act as organic transporters of bacterial bioactive compounds [10]. The diverse bioactivities and functionalities of probiotic-derived extracellular vesicles (EVs) are continually being discovered and utilized. For example, the commensal *Escherichia coli* strains EcN and ECOR63 create outer membrane vesicles (OMVs) that improve epithelial barrier function by upregulating ZO-1 and claudin-14 expression levels while simultaneously reducing claudin-2 expression [11]. Microvesicles (MVs) derived from *Limosilactobacillus kefiri*, *Limosilactobacillus kefiranofaciens*, and *Limosilactobacillus kefirgranum*, species native to kefir grains, may demonstrate anti-inflammatory properties by suppressing the synthesis of pro-inflammatory cytokines, including IL-8 and TNF-α, via the NF-κB signalling pathway. Furthermore, these MVs have been shown to inhibit intestinal hemorrhage and diarrhea in a trinitrobenzene sulfonic acid (TNBS)-induced experimental colitis murine model [12].

*A. muciniphila* produces extracellular vesicles (Am-EVs), similar to other bacteria. Emerging evidence suggests that these nanoscale vesicles play a critical role in host-microbiota interactions, particularly in the pathogenesis of inflammatory bowel disease (IBD). Kang et al. [13] demonstrated that mice treated with Am-EVs exhibited reduced weight loss, increased colon length, and lower disease activity index (DAI) scores, indicating alleviation of colitis symptoms. Additionally, histological assessments revealed improved colonic epithelial stability and reduced inflammatory cell infiltration in the colonic mucosa of these mice. In a separate study, Chelakkot et al. [14] found that Am-EVs were negatively correlated with the severity of ulcerative colitis (UC). In a murine UC model, Am-EVs improved intestinal barrier function by regulating tight junction protein expression, reducing pathogen-induced cytotoxicity, and suppressing inflammatory responses. Despite these promising findings, the precise mechanisms by which Am-EVs alleviate intestinal inflammation remain unclear, warranting further investigation.

Given that the intestine serves as a site for microbial communities to interact with the host and that intestinal epithelial cells constitute the primary cellular lineage responsible for forming the mechanical barrier and secreting mucins, they play a crucial role in maintaining intestinal homeostasis. To study the effects of functional substances on gut inflammation, the lipopolysaccharide (LPS)-induced inflammation model in Caco-2 cells has been widely utilized [15,16]. In this paper, we decided to investigate the potential anti-inflammatory effects of *Akkermansia muciniphila*-derived extracellular vesicles (Am-EVs) on LPS-induced inflammation in Caco-2 cells, with the aim of elucidating their molecular mechanisms and advancing our understanding of gut microbiota function–axis interactions.

## 2. Materials and Methods

### 2.1. Preparation of A. muciniphila

The *A. muciniphila* MucT (ATCC BAA-835) was purchased from the Shanghai Biological Specimen Conservation Centre, Shanghai, China. Cultivation was conducted under anaerobic conditions at 37 °C in brain heart infusion (BHI) broth supplemented with 0.5% mucin (TypeIII, Sigma, St. Louis, MO, USA).

### 2.2. Isolation and Characterization of A. muciniphila-Derived Extracellular Vesicles

The bacterial suspension was centrifuged at 4410× *g* for 10 min at 25 °C. The resulting supernatant was filtered through a 0.45 μm filter (aqueous phase) and transferred to sterilized ultracentrifuge tubes. The samples were then centrifuged at 120,000× *g* for 70 min at 4 °C (the ultracentrifuge Optima XPN is manufactured by Beckman Coulter International Trade Co., Ltd., Shanghai, China). The supernatant was discarded, and the pellet was resuspended in a small volume of sterile PBS buffer (pH is 7.4). This centrifugation step was repeated twice to wash the pellet. Finally, the pellet was weighed and resuspended in 1 mL of PBS, yielding a concentrated preparation of the Am-EVs, which was stored at −80 °C for future use. The morphology of the isolated EVs was observed using the JEM-1200EX cryo-transmission electron microscope (JEOL, Tokyo, Japan). The particle size of the Am-EVs was determined using a ZS90 Malvern nanoparticle size analyzer (Malvern Panalytical, Malvern, UK). The protein concentration was initially quantified using a BCA protein assay kit (Beyotime Biotechnology, Beijing, China) and protein molecular weight sizes were determined using sodium dodecyl sulfate polyacrylamide gel electrophoresis (SDS-PAGE).

### 2.3. Protein Sequence Identification of A. muciniphila-Derived Extracellular Vesicles

The Am-EVs were reacted with 5 mM DTT (Amresco, Solon, OH, USA) at 37 °C for 1 h, and 10 mM IAM (Amresco, Solon, OH, USA) was added for 45 min at room temperature without light. The sample was diluted 4 times with 25 mM ammonium bicarbonate and digested with trypsin. After the overnight reaction at 37 °C, formic acid was used to adjust the pH to less than 3 to terminate the enzyme digestion reaction. The samples were desalted using a C18 desalting column, the flow fluid was collected, and the samples were freeze-dried in a LGJ-1C-56 freeze-drying machine (Beijing Yataikelong Instrument Technology Co., Ltd., Beijing, China). The solution was dissolved with 10 µL of 0.1% formic acid aqueous solution, centrifuged at 14,000× *g* at 4 °C for 20 min, and the supernatant was added to the sample; the elution gradient is shown in Table 1. The supernatant was subject to high-performance liquid chromatography–tandem mass spectrometry (HPLC-MS) analysis to identify its protein sequence, using a Q-Exactive HF-X mass spectrometer (Thermo Fisher Scientific, Bremen, Germany) equipped with a Nanospray Flex™ (NSI) ion source (Thermo Fisher Scientific, San Jose, CA, USA).

For the separation, the mobile phase A was water with 0.1% formic acid, and mobile phase B was 80% aqueous acetonitrile with 0.1% formic acid. The HPLC-MS conditions were the nanospray voltage was set to 2.2 kV, and the heated capillary temperature was maintained at 320 °C. The data-dependent acquisition (DDA) mode was employed for mass spectrometry, with a full scan mass range of *m*/*z* 350–1500. The resolution for the first-stage mass analysis was set to 120,000 at *m*/*z* 200, with an automatic gain control (AGC) target of 3 × 10^6^ and a maximum injection time of 80 ms for the C-trap. The 40 most intense precursor ions from the survey scan were selected for high-energy collisional dissociation (HCD)-based fragmentation. The resolution for the subsequent tandem mass spectrometry (MS/MS) analysis was set to 15,000 at *m*/*z* 200, with an AGC target of 5 × 10^4^ and a maximum injection time of 45 ms. The collision energy for peptide fragmentation was set at 27%.

### 2.4. Proteomic Analysis

The protein sequences in FASTA format were input into the Cello2GO web server. The subcellular localization of the target protein sequences was predicted using the Cello2GO online tool (http://cello.life.nctu.edu.tw/cello2go/ (accessed on 21 November 2024)) [17]. GO and KEGG enrichment analyses were performed on proteins in the library search results [18,19]; GO annotation information was obtained from the GO database (https://www.ebi.ac.uk/QuickGO/ (accessed on 12 December 2024)); KEGG annotations were retrieved from the KEGG database (http://www.kegg.jp/kegg/pathway.html (accessed on 12 December 2024)). The statistical significance of protein enrichment in KEGG pathways was determined using the hypergeometric distribution test to calculate *p*-values [20]. Enrichment bar graphs were generated using ggplot2 in R language [21].

### 2.5. Cell Culture

Caco-2 cells (obtained from Fu Heng BioLogy, Shanghai, China) were maintained in RPMI 1640 medium (EallBio Biomedical Technology, Beijing, China) enriched with 15% FBS (Fu Heng BioLogy, Shanghai, China) and 1% penicillin-streptomycin (EallBio Biomedical Technology, Beijing, China). The cells were seeded into T25 (NEST, Hangzhou, China) culture flasks and incubated at 37 °C within a humidified atmosphere containing 5% CO_2_ [22]. The cells were allowed to grow for about 3 days until complete adherence to the flask surface was achieved and the confluence reached approximately 80%, at which point they were utilized for subsequent experimental procedures.

### 2.6. Cell Viability Assay

Caco-2 cells in logarithmic phase were seeded into 96-well plates at a density of 5 × 10^5^ cells/mL, with 100 μL per well. To maintain humidity, 200 μL of PBS was added to the periphery of each well. Once the cells reached 80% confluence, the medium was removed. The cells were then treated with serum-free RPMI 1640 medium containing various concentrations of Am-EVs (0, 1, 10, 20, 30, 40, 50, and 100 μg/mL) and LPS (0.1, 1, 10, and 100 μg/mL) for 24 h to determine the optimal concentrations. To assess the effects of Am-EVs and LPS on cell viability, the MTT assay kit (Beyotime Biotechnology Co., Ltd., Beijing, China) was used according to the manufacturer’s instructions. Cells were pre-treated with 10 μg/mL LPS in serum-free medium for 18 h. After removing the supernatant, various concentrations of Am-EVs (0, 1, 25, and 50 μg/mL) were added for an additional 12 h. Following incubation, the medium was replaced with fresh medium and 10 μL of MTT solution per well and incubated for 4 h. Then, 100 μL of SDS solution was added to each well and incubated for 12 h. Cell viability was determined by measuring the absorbance at 570 nm using a microplate reader, with calculations based on the specified Formula (1).

(1) $$\mathrm{Cell}\;\mathrm{Viability}\;\left(\%\right)=\left({\mathrm{OD}}_{\mathrm{Sample}}-{\mathrm{OD}}_{\mathrm{Blank}}\right)/\left({\mathrm{OD}}_{\mathrm{Control}}-{\mathrm{OD}}_{\mathrm{Blank}}\right)\;\times \;100\%$$

### 2.7. Cell Treatment

Caco-2 cells, cultured in the logarithmic phase, were seeded into 96-well plates at 5 × 10^5^ cells/mL, with 100 μL of cell suspension per well. To maintain humidity, 200 μL of PBS was added to the periphery of each well. Upon reaching 80% confluence, the medium was removed, and the cells were treated with 100 μL of serum-free medium containing 10 μg/mL LPS for 18 h. After discarding the medium, various concentrations of Am-EVs (1, 25, and 50 μg/mL) were added in 100 μL volumes and incubated for an additional 12 h. These cells were prepared for subsequent NO and ROS experiments.

Caco-2 cells, in the logarithmic phase of growth, were seeded into 35 mm dishes (NEST, Hangzhou, China) at a concentration of 6 × 10^5^ cells/mL, with 3 mL of the cell suspension aliquoted into each well. At 80% confluence, the medium was replaced with 3 mL of serum-free medium containing 10 μg/mL LPS for 18 h. After medium removal, 3 mL of Am-EVs at various concentrations (1, 25, and 50 μg/mL) were added and incubated for another 12 h. These cells were prepared for subsequent MDA, CAT, qRT-PCR, transcriptome analysis, and Western blot experiments.

### 2.8. Determination of Nitric Oxide (NO) and Reactive Oxygen Species (ROS) Levels

The cell-culture treatment is shown in Section 2.7; following incubation, supernatants were collected for NO quantification using the Total NO assay kit (Beyotime Biotechnology, Beijing, China) and refer to the instruction manual for experimental procedures. For ROS measurement, the ROS assay kit (Beyotime Biotechnology Co., Ltd., Beijing, China) was used according to the manufacturer’s instructions; cells were treated with 10 μmol/mL DCFH-DA in replacement medium and incubated at 37 °C with 5% CO_2_ for 30 min. Afterward, the medium was aspirated, wells were washed twice with PBS, and 200 μL of serum-free medium was added to each well. Fluorescence intensity was determined using a spectrophotometer at an excitation wavelength of 485 nm and an emission wavelength of 525 nm (Tecan, Männedorf, Switzerland).

### 2.9. Determination of Malondialdehyde (MDA) and Catalase (CAT) Levels

The cell-culture treatment is shown in Section 2.7; post-cultivation, cells were rinsed thrice with PBS buffer and lysed on ice for 5–8 min using Beyotime’s Western and IP lysis buffer. Cell lysates were harvested with a cell scraper and centrifuged at 12,000× *g* at 4 °C for 10 min to separate the supernatant. Protein concentrations were assayed to normalize MDA and CAT levels. The activities of MDA and CAT were measured using assay kits (Beyotime Biotechnology, Beijing, China), following the experimental procedures provided by the manufacturer.

### 2.10. qRT-PCR Analysis

Cells were treated as in Section 2.7 and then washed three times with PBS buffer at the end of the culture. Total RNA extraction from Caco-2 cells was conducted using Trizol reagent, following the supplier’s instructions. Reverse transcription was carried out with the revertAce qPCR RT master mix, which includes gDNA remover (Toyobo, Osaka, Japan). For RT-PCR, approximately 0.5 μg of total RNA was utilized on a Bio-Rad CFX96 Touch system with SYBR Green master mix (Toyobo, Osaka, Japan). The expression levels of TNF-α, IL-1β, and IL-6 were determined relative to the endogenous control β-actin. Primer sequences are detailed in Table 2. The qRT-PCR protocol consisted of an initial denaturation step at 95 °C for 2 min, followed by 38 cycles of 95 °C for 30 s, 55 °C for 30 s, and 72 °C for 30 s, concluding with a final extension at 72 °C for 2 min.

### 2.11. Transcriptome Analysis and Key Protein Interactions

Cell-spreading dishes as depicted in Section 2.7. The treatment group was cultured with 3 mL of serum-free medium supplemented with 10 μg/mL LPS for 18 h, followed by the addition of 3 mL of 50 μg/mL Am-EVs for a further 12 h under identical conditions. In a biosafety cabinet, the supernatant was removed, and Caco-2 cells were washed thrice with PBS. Each dish was treated with 1 mL TRIzol reagent for 5 min to lyse the cells, followed by homogenization using a pipette. The resulting lysate was transferred to 1.5 mL RNase-free EP tubes and stored at −80 °C prior to sequencing analysis.

Using synthesis-by-sequencing (SBS) technology on the Illumina sequencing platform, we sequenced the cDNA library with base quality scores reaching or exceeding Q30. Differential gene expression analysis was performed using edgeR software (Version 4.0) to identify differentially expressed genes (DEGs) between groups, yielding DEG sets under two biological states. DEGs were filtered with a fold change ≥1.5 and *p*-value ≤ 0.05. False discovery rates (FDRs) were calculated following *p*-value adjustment. A volcano plot was created to observe the difference in gene expression levels in two groups, as well as the statistical significance [23]. Pathway significant enrichment analysis was conducted by taking the pathway in the KEGG database as the unit to find pathways that are significantly enriched in DEGs compared with the background of the entire genome [24]. Combining the results of differential expression analysis and the STRING database, we identified the interaction network and analyzed it with Cytoscape (Version 3.10.3) for the major core proteins [25]. Pathway enrichment analysis was then conducted with the KEGG database to discern significantly enriched pathways among the DEGs relative to the genome-wide background.

### 2.12. Western Blot

The cell culture was treated as in Section 2.7 and then washed three times with a PBS buffer at the end of the culture. Then, cells were harvested using a cell scraper and lysed in an ice-cold lysis buffer supplemented with phosphatase and protease inhibitors. After 45 min on ice, the lysates were centrifuged at 1500× *g* to obtain supernatants. The extracted proteins were mixed 3:1 with 4× DDT (Biorigin, Shanghai, China) and the mixture was denatured by boiling in a water bath at 100 °C for 15 min. Protein concentrations were determined using the BCA Protein Assay Kit (Beyotime Biotechnology, Beijing, China). Equal amounts of protein (30 μg) from each sample were resolved by SDS-PAGE and electrotransferred onto PVDF membranes (Merck KGaA, Darmstadt, Germany). Membranes were blocked with 5% non-fat milk in TBST for 2 h, incubated with primary antibodies overnight at 4 °C, and then washed three times with TBST. After incubation with secondary antibodies for 2 h at room temperature and subsequent TBST washes, immunoreactive bands were visualized using Tanon™ High-sig ECL Western Blotting Substrate (Tanon, Shanghai, China) and quantified using the ImageJ software (Version 1.52v, National Institutes of Health, Bethesda, MD, USA).

### 2.13. Statistical Analysis

All experiments were performed in triplicate. Results are expressed as the mean ± standard deviation. Statistical comparisons were conducted using one-way analysis of variance (ANOVA) followed by the Waller–Duncan post-hoc test in the SPSS (IBM corp. Version 21, Armonk, NY, USA) statistics software. A *p*-value of less than 0.05 was considered statistically significant. Graphs were plotted using GraphPad Prism software (Version GraphPad Software 9, Boston, MA, USA).

## 3. Results

### 3.1. The Preparation and Characterization of Am-EVs

**Figure 1:**
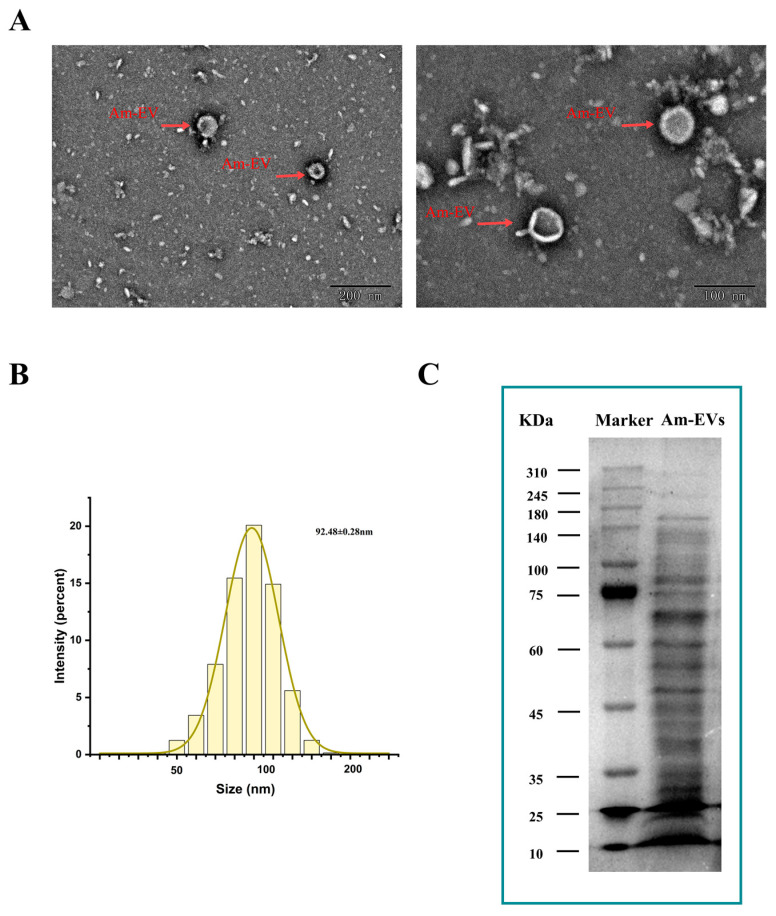
Characterization and identification of Am-EVs. (**A**) Morphological characteristics of Am-EVs observed by transmission electron microscopy; (**B**) particle size analysis and identification of Am-EVs; and (**C**) proteins of Am-EVs in the SDS-PAGE. (M is a standard protein marker with a measurement range of 10–310 kDa).

Vesicles released by *A. muciniphila* were clearly visible using transmission electron microscopy. They had sizes ranging from 50 to 200 nm and displayed distinctive double-membrane architectures (Figure 1A). With an average particle diameter of 92.48 ± 0.28 nm, the size distribution of Am-EVs followed a normal distribution (Figure 1B). According to the BCA protein assay, the concentration of Am-EVs was 14.50 ± 2.23 mg/mL. Additional examination of Am-EVs’ protein composition using SDS-PAGE electrophoresis showed a wide range of protein bands with molecular weights between 10 and 240 kDa, as well as a comparatively high protein content in the area around 70 kDa (Figure 1C).

### 3.2. Protein Identification of Am-EVs

**Figure 2:**
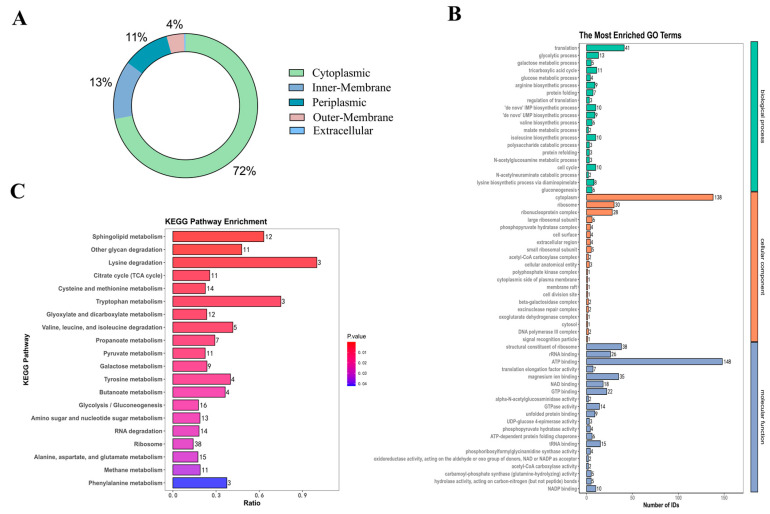
Protein identification of Am-EVs. (**A**) Mapping of identified subcellular structures; (**B**) Am-EVs total-protein GO analysis; (**C**) Am-EVs total-protein qualitative KEGG analysis.

HPLC-MS was used in this investigation to identify 850 proteins in Am-EVs. There were 546 unknown proteins, according to additional subcellular localization analysis of the proteins found in Am-EVs. The remaining 304 proteins were split into three groups, with 219 cytoplasmic proteins making up the largest proportion (72%), 40 inner-membrane proteins (13%), 32 periplasmic proteins (11%), 12 outer-membrane proteins (4%), and only 1 extracellular protein as the smallest percentage (Figure 2A).

Proteins within Am-EVs underwent gene ontology (GO) analysis, categorizing them into biological processes (BP), cellular components (CC), and molecular functions (MF) as depicted in Figure 2B. The BP analysis showed a significant enrichment of Am-EV proteins in processes such as translation, glycolytic process, and fatty acid biosynthesis. The CC analysis indicated that these proteins were predominantly found in the cytoplasm, ribosome, and ribonucleoprotein complex. The MF analysis revealed that the key molecular functions of Am-EV proteins were ATP binding, the structural constituent of ribosome, and magnesium ion binding.

Through KEGG analysis (Figure 2C), we further investigated the pathways involved in Am-EV proteins. The results indicated that Am-EV proteins are predominantly engaged in sphingolipid metabolism, degradation of other polysaccharides, β-lactam resistance, lysine degradation, glyoxylate and dicarboxylate metabolism, cysteine and methionine metabolism, tryptophan metabolism, phosphotransferase system (PTS), degradation of valine, leucine, and isoleucine, ribosome function, tyrosine metabolism, citrate cycle (TCA cycle), and galactose metabolism, among other biological processes.

### 3.3. Am-EVs Alleviated LPS-Induced Cytotoxicity in Caco-2 Cells

In this experiment, a model of inflammation was constructed by inducing Caco-2 cells with 10 μg/mL of LPS for 18 h (Supplementary Figure S1).

With the increase in Am-EVs concentration (1–100 μg/mL), the viability of Caco-2 cells gradually increased (Supplementary Figure S2A), indicating that Am-EVs are not cytotoxic and may even promote cell proliferation under these conditions. Concentrations of 1, 25, and 50 μg/mL Am-EVs were selected for subsequent experiments. When Caco-2 cells were stimulated with 10 μg/mL LPS for 18 h and then co-cultured with varying concentrations (1, 25, and 50 μg/mL) of Am-EVs for an additional 12 h, the cell viability continued to increase with the concentration of Am-EVs (Supplementary Figure S2B). In summary, concentrations of 1, 25, and 50 μg/mL Am-EVs were chosen for further experimentation.

### 3.4. Am-EVs Ameliorated the LPS-Induced Oxidative Stress in Caco-2 Cells

**Figure 3:**
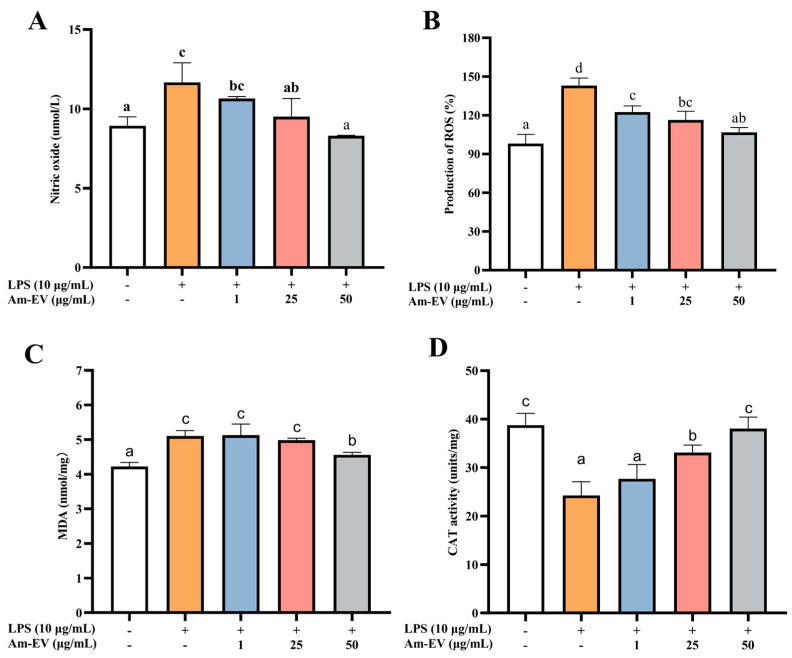
Effect of Am-EVs on LPS-induced generation of oxidative stress. (**A**) Effects of Am-EVs on the production of NO; (**B**) effects of Am-EVs on the production of ROS; (**C**) Am-EVs on the production of MDA; and (**D**) effects of Am-EVs on the production of CAT. Note: Different letters indicate significant differences (*p* < 0.05).

Nitric oxide (NO) is a free radical species that serves as a key contributor to the initiation and progression of oxidative stress within biological systems. As depicted in Figure 3A, following induction with LPS in Caco-2 cells, the concentration of NO exhibited a significant upward trend compared to the control group (*p* < 0.05). Upon the addition of various concentrations of Am-EVs, a gradual decrease in NO content was observed, which was associated with an increase in Am-EVs concentration, demonstrating a dose-dependent effect. Notably, Am-EVs at a concentration of 50 μg/mL exhibited the most pronounced inhibitory effect on NO.

In Figure 3B, treatment with LPS at 10 μg/mL significantly elevated reactive oxygen species (ROS) levels compared to the control (*p* < 0.05). The addition of Am-EVs at concentrations of 1, 25, and 50 μg/mL led to a concentration-dependent decrease in ROS content. Malondialdehyde (MDA) is a well-established marker of lipid peroxidation, commonly used to assess cellular oxidative stress. Figure 3C shows that LPS induction significantly increased MDA levels (*p* < 0.05), while Am-EVs treatment for 12 h reduced MDA levels in a dose-dependent manner. Additionally, LPS stimulation decreased catalase (CAT) release in Caco-2 cells (*p* < 0.05), as shown in Figure 3D. Am-EVs treatment increased CAT activity in a dose-dependent manner, with concentrations of 25 μg/mL and 50 μg/mL significantly enhancing CAT activity (*p* < 0.05). Notably, the 50 μg/mL Am-EVs treatment restored CAT activity to levels comparable to the blank control group.

### 3.5. Am-EVs Ameliorated the LPS-Induced Inflammation in Caco-2 Cells

**Figure 4:**
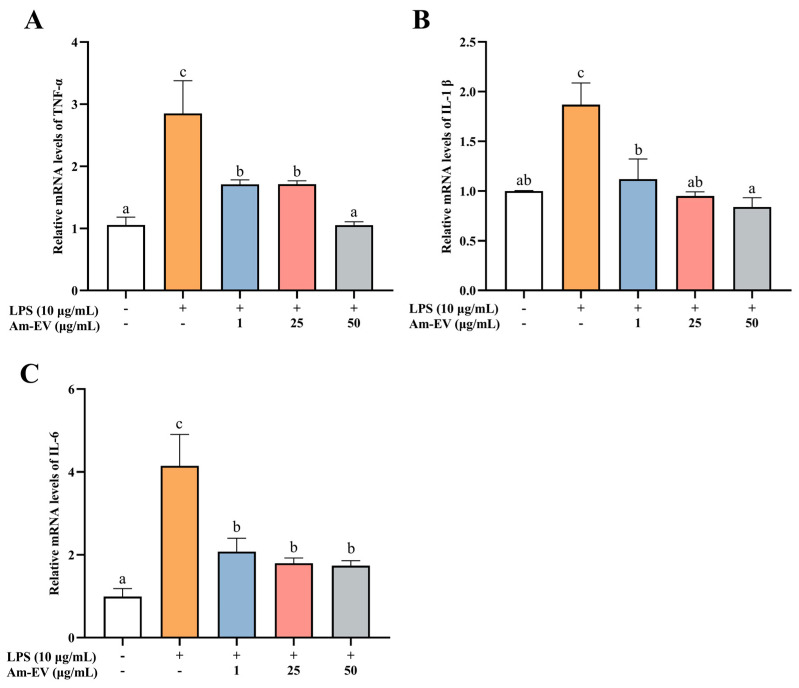
Effect of Am-EVs on LPS-induced expression of inflammatory factors. (**A**) TNF-α; (**B**) IL-1β; and (**C**) IL-6. Note: Different letters indicate significant differences (*p* < 0.05).

Tumor necrosis factor alpha (TNF-α), interleukin-1β (IL-1β), and interleukin-6 (IL-6) are pro-inflammatory cytokines that are commonly observed in biological systems. As shown in Figure 4, 10 μg/mL of LPS significantly induced the upregulation of TNF-α, IL-1β, and IL-6 mRNA expression (*p* < 0.05). Various concentrations of Am-EVs (1, 25, and 50 μg/mL) suppressed the release of inflammatory cytokines in LPS-induced Caco-2 cells in a dose-dependent manner. Notably, pretreatment with 50 μg/mL Am-EVs reduced the mRNA expression levels of TNF-α, IL-1β, and IL-6 compared to LPS-treated cells (*p* < 0.05). These findings indicate that Am-EVs have a superior inhibitory effect on the level of LPS-induced intestinal inflammation.

### 3.6. Am-EVs Through the MAPK Signaling Pathway Regulate LPS-Induced Inflammation in Caco-2 Cells in RNA Sequencing Analysis

**Figure 5:**
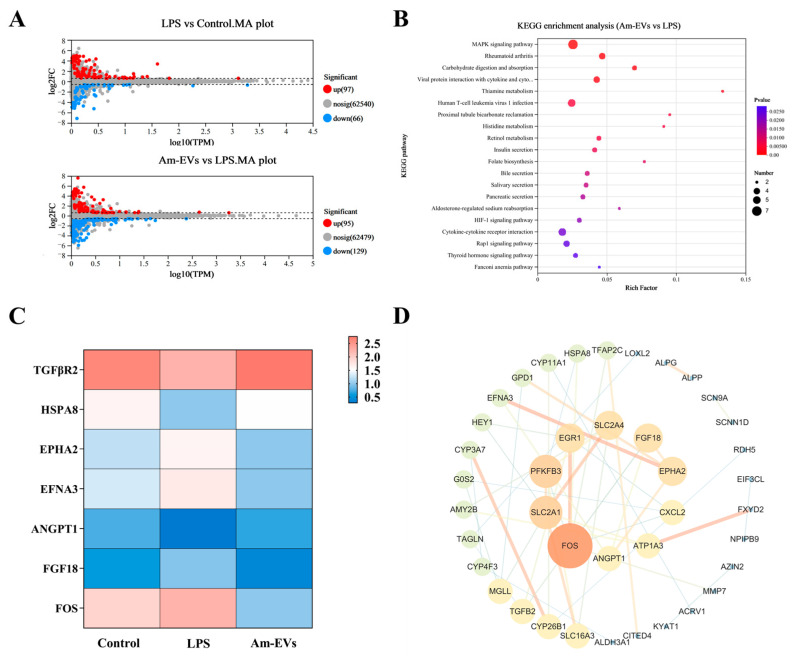
Transcriptome sequencing. (**A**) Differential gene MA plots for LPS vs. control and Am-EVs vs. LPS; (**B**) KEGG enrichment analysis plots for Am-EVs vs. LPS; (**C**) differentially expressed genes related to the MAPK signaling pathway; and (**D**) PPI of Am-EVs with LPS differential genes.

Statistical analysis was performed on the differentially expressed genes among groups, and the results were visualized using volcano plots to represent the number of differentially expressed genes between different groups (Figure 5A). Compared to the control group, treatment of Caco-2 cells with 10 μg/mL LPS upregulated 97 genes and downregulated 66 genes. In contrast, compared to the LPS group, the 50 μg/mL Am-EVs group downregulated 129 genes and upregulated 95 genes (*p* ≤ 0.05; fold change = 1.5).

In our study, differential gene enrichment analysis using the KEGG database revealed that genes differentially expressed between the Am-EVs and LPS-induced groups were significantly enriched in pathways such as the MAPK signaling pathway, rheumatoid arthritis signaling pathway, carbohydrate digestion and absorption, and interactions between viral proteins and host factors including cytokines and their receptors (*p* ≤ 0.05, Figure 5B). The results showed (Figure 5C) that the proteins on the first signaling pathway MAPK were TGFBR2, HSPA8, ANGPT1, EPHA2, EFNA3, FGF18, and FOS. Further analysis showed that TGFBR2, HSPA8, and ANGPT1 genes in LPS group were significantly downregulated compared with the control group (*p* ≤ 0.05), and these genes in the Am-EVs group were significantly upregulated compared with the LPS group (*p* < 0.05). EPHA2, EFNA3, FGF18, and FOS genes in the LPS group were significantly upregulated compared with the control group (*p* ≤ 0.05), and these genes were significantly downregulated in the Am-EVs group compared with the LPS group. The results of the protein–protein interaction (PPI) network between Am-EV and lipopolysaccharide (LPS) treatment showed that the top 10 core proteins were identified as FOS, SLC2A1, PFKFB3, EGR1, SLC2A4, FGF18, EPHA2, CXCL2, ATP1A3, and ANGPT1 (Figure 5D), among which four were all proteins on the MAPK pathway.

### 3.7. Am-EVs Modulate the Expression of MAPK Pathway-Related Genes to Inhibit LPS-Induced Inflammation in Caco-2 Cells in WB Analysis

**Figure 6:**
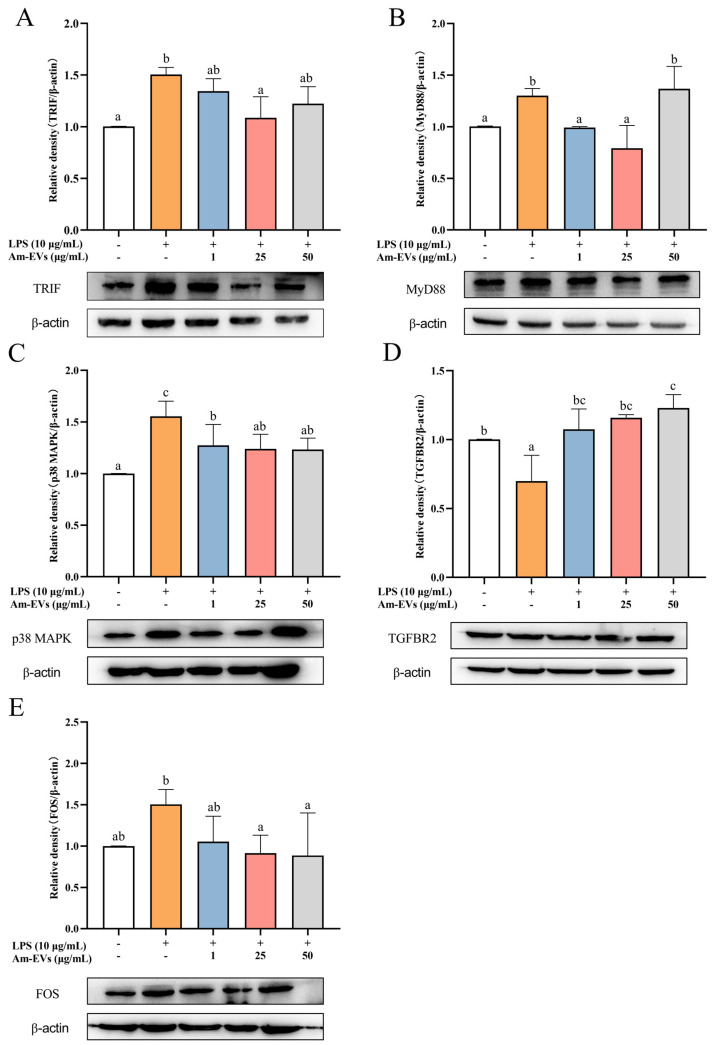
Effects of Am-EV1 on protein expression that are involved in the MAPK inflammatory pathway in LPS-stimulated Caco-2 cells. (**A**) MyD88; (**B**) TRIF; (**C**) p38 MAPK; (**D**) TGFBR2; and (**E**) FOS. Note: Different letters indicate significant differences (*p* < 0.05).

Combined with the results of transcriptome sequencing, this study examined the changes of TRIF, MyD88, p38 MAPK, FOS, and TGFBR2 protein expression in LPS-stimulated Caco-2 cells using the Western blot technique. The results, shown in Figure 6, demonstrate that TRIF, MyD88, p38 MAPK, and FOS protein expression was significantly increased, while TGFBR2 expression was significantly decreased (*p* < 0.05) in Caco-2 cells after LPS stimulation compared with the normal control. In addition, Am-EVs treatment significantly decreased TRIF, MyD88, p38 MAPK, and FOS protein levels and increased TGFBR2 expression in Caco-2 cells.

## 4. Discussion

A substantial body of research has documented the crucial functions and mechanisms of bacterial-derived extracellular vesicles (EVs) in the treatment of various diseases; however, studies on EVs derived from *A. muciniphila* are still in their infancy. In this study, EVs were isolated from *A. muciniphila* by ultra-high-speed centrifugation and were spherical in shape with a vesicular structure; their diameter was 92.48 ± 0.28 nm, and the outermost layer was a lipid bilayer structure. These results are consistent with the morphological characteristics captured by Chelakkot et al. [14] using transmission electron microscopy (TEM), which depicted the EVs as irregularly spherical vesicles.

The content of extracellular vesicles (EVs) varies significantly among different bacterial origins, and even within the same bacterial strain, the EV content can differ under various culture conditions and extraction methods [26,27]. Lee et al. [28] reported that *Staphylococcus aureus*-derived EVs (Sa-EVs) contain 90 proteins, predominantly cytoplasmic. A subsequent study by Tartaglia et al. [29] identified over 200 proteins associated with Sa-EVs, with 160 being cytoplasmic proteins. Regarding the origin of EVs, the proteins they carry can be found both inside the vesicles and on the EV membranes. For example, outer membrane vesicles (OMVs) contain periplasmic components such as lipoproteins, lipids, and outer membrane proteins, while inner outer membrane vesicles (IOMVs) include cytoplasmic and periplasmic membrane components and are enriched with ATPs and DNA [30]. In our study, we analyzed the proteome of Am-EVs using HPLC-MS, identifying 850 distinct proteins. Subsequent subcellular localization analysis revealed that Am-EVs mainly carry cytoplasmic proteins, with 219 proteins identified as such. These results align with previous subcellular localization studies of EVs from other Gram-negative bacteria, suggesting that Am-EVs primarily transport cytoplasmic proteins [31].

The gene ontology (GO) analysis of proteins within Am-EVs revealed key processes such as translation, glycolysis, and fatty acid biosynthesis, which are crucial for cellular metabolism and energy production. These processes are often linked to antioxidant defense mechanisms, as proper metabolic activity is essential for reducing the buildup of reactive oxygen species (ROS) that contribute to oxidative stress [32]. Furthermore, enzymes involved in glycolysis and fatty acid biosynthesis are known to be modulated under oxidative stress conditions, helping cells manage redox balance [33,34]. The KEGG pathway analysis sheds light on specific pathways that Am-EV proteins engage in, many of which are linked to metabolic and stress-response functions. For instance, the involvement of proteins in sphingolipid metabolism and β-lactam resistance suggests roles in cellular protection and stress response [35,36]. Sphingolipids, which have been implicated in regulating cellular responses to stress and inflammation, may contribute to Am-EVs’ anti-inflammatory effects. The pathways involved in cysteine and methionine metabolism, along with tryptophan metabolism, are known to impact oxidative stress regulation and inflammatory responses [37,38,39]. The citrate cycle (TCA cycle), which plays a central role in energy production, is also integral to cellular responses under stress [40], suggesting that Am-EVs might help restore metabolic homeostasis during oxidative or inflammatory challenges. Overall, the functional characterization of Am-EV proteins underscores their key role in oxidative stress defense and anti-inflammatory mechanisms, which is consistent with our experimental results.

In recent years, extracellular vesicles (EVs) derived from probiotics have demonstrated significant effects in modulating inflammatory responses. For instance, EVs released by *Bacteroides fragilis* have induced the secretion of anti-inflammatory cytokines while reducing the secretion of pro-inflammatory cytokines [41]. Furthermore, they have successfully suppressed inflammatory responses in DSS-induced colitis models through the activation of regulatory T cells (Treg) [42]. Extracellular vesicles (EVs) from *Lactobacillus rhamnosus* have been shown to induce increased production of the anti-inflammatory cytokine IL-10 in the mesenteric and Peyer’s patches of mice while enhancing the response of regulatory T cells (Treg), thereby contributing to the modulation of immune system balance and suppression of inflammatory responses [12]. EVs derived from *Lactobacillus paracasei* effectively reduce the mRNA expression levels of pro-inflammatory cytokines IL-1α, IL-1β, IL-2, and TNF-α, and significantly upregulate the mRNA expression of anti-inflammatory cytokines IL-10 and TGF-β. Additionally, these EVs have demonstrated an inhibitory effect on the expression of inflammation-associated proteins such as COX-2, iNOS, and NF-κB, and have suppressed the activation of NO [43]. Our results showed that both 25 and 50 μg/mL of Am-EVs were able to reduce intracellular NO, ROS, and MDA content and elevate CAT enzyme activity to a certain extent, thereby attenuating oxidative stress damage in Caco-2 cells, as well as decreasing the mRNA expression levels of inflammatory factors TNF-α, IL-1β, and IL-6. Compared to other studies, this investigation delved into the protective effects of Am-EVs on intestinal inflammation through in vitro experiments and explored their underlying mechanisms. Notice that this study employs a Caco-2 cell model for experimentation, which can only simulate partial pharmacokinetic behavior within the human body. Compared to an animal model, the Caco-2 cells lack the digestive system’s role, leading to potential discrepancies in drug absorption and metabolism observed in vivo or in a more complex environment such as the body of an animal subject. Additionally, within an animal model, due to the intricate physiological conditions (including the involvement of the digestive system), further insights into the dynamic effects of drugs can be revealed.

Combined with PPI and KEGG enrichment pathway analyses, the core proteins FOS, FGF18, EPHA2, and ANGPT1 in the inflammatory response of Am-EVs and LPS-induced Caco-2 cells were all related proteins on the major pathway MAPK, as shown by the KEGG enrichment results, and FOS was the most prominent core protein. Therefore, we suggest that the MAPK signaling pathway may be the main pathway by which Am-EVs inhibit LPS-induced inflammation in Caco-2 cells. The MAPK signaling pathway, an eukaryotic cellular signaling module, can be activated under various stimuli, such as environmental stress, cytokines, and microbial products, thereby regulating cell growth, differentiation, migration, and inflammation [44]. We found that, among the MAPK-related genes, the upstream signal of the MAPK pathway, the FOS gene, was significantly downregulated and the downstream signal, the TGFBR2 gene, was significantly increased in the Am-EVs-treated group. The western blot results further showed that Am-EVs significantly decreased the protein levels of TRIF, MyD88, p38 MAPK, and FOS and increased the TGFBR2 expression in Caco-2 cells. The downregulation of FOS may play a regulatory role in the cell cycle and cellular stress responses [45], which is consistent with our experimental results in Section 3.4. TGFBR2 is a key receptor in the TGF-β signaling pathway, and its upregulation may indicate that Am-EVs play a role in regulating extracellular matrix remodeling, cell proliferation, and differentiation [46]. This reveals that while influencing the MAPK signaling pathway, Am-EVs may also comprehensively regulate cellular behavior through interactions with other signaling pathways, such as the TGF-β signaling pathway. Collectively, Am-EVs may impact the MAPK signaling pathway by decreasing the protein levels of TRIF and MyD88. TRIF and MyD88 are key adapter proteins in the toll-like receptor (TLR) signaling pathways, which, upon recognition of pathogen-associated molecular patterns (PAMPs), activate downstream signaling molecules, including members of the MAPK family [47]. As one of the pathways most closely associated with inflammatory bowel disease, the MAPK signaling pathway activates the transcription factors FOS and C-Jun through the sequential activation of p38, ERK, and JNK, promoting their translocation to the nucleus to form the transcription factor AP-1, which regulates the transcription of inflammatory factors such as IL-6 and TNF-α [48]. Therefore, the reduction in the expression level of these proteins by Am-EV indicates that they may inhibit TLRs-mediated inflammatory signal transduction, thereby reducing the activation of p38 MAPK, and down-regulating the activity of the AP-1 transcription factor by FOS, thereby reducing the expression of inflammation-related genes and affecting the MAPK pathway. Future studies should further elucidate the precise molecular interactions between Am-EVs and these signaling molecules to validate their therapeutic potential.

## 5. Conclusions

Through this study, we successfully extracted and characterized extracellular vesicles (Am-EVs) derived from *Akkermansia muciniphila* and revealed their molecular mechanisms in anti-inflammatory effects. Am-EVs exhibited significant anti-inflammatory properties, reducing oxidative stress markers (such as ROS, NO, and MDA) and the expression levels of pro-inflammatory factors (TNF-α, IL-1β, and IL-6) in vitro LPS-induced inflammation models, while restoring CAT activity. Transcriptomic analysis and Western blot experiments further confirmed that the anti-inflammatory activity of Am-EVs is related to the MAPK signaling pathway. Am-EVs achieve anti-inflammatory functions by downregulating the expression of key genes such as TRIF, MyD88, p38 MAPK, and FOS, and upregulating the expression of TGFBR2. These findings provide important evidence for understanding the unique mechanisms by which *A. muciniphila* regulates the host immune system through EVs and reveal its potential therapeutic value in intestinal diseases such as inflammatory bowel disease (IBD). Future research can further explore the application potential of Am-EVs in in vivo models, especially in terms of therapeutic effects and safety in intestinal inflammatory diseases like IBD.

## Figures and Tables

**Table 1:** Gradient elution procedure.

Time/min	Mobile Phase A	Mobile Phase B
0	92	8
5	88	12
35	70	30
44	60	40
45	5	95
60	5	95

**Table 2:** Primer sequences.

Primer Name	Primer Sequence
IL-6-F	CACTGGTCTTTTGGAGTTTGAG
IL-6-R	GGACTTTTGTACTCATCTGCAC
TNF-α-F	TGGCGTGGAGCTGAGAGATAACC
TNF-α-R	GACGGCGATGCGGCTGATG
IL-1β-R	CAGTGGCAATGAGGATGACTTGTTC
IL-1β-F	CTGTAGTGGTGGTCGGAGATTCG
β-actin-F	CCTAGAAGCATTTGCGGTGCACGATG
β-actin-R	TCATGAAGTGTGACGTTGACATCCGT

## Data Availability

The original contributions presented in the study are included in the article, further inquiries can be directed to the corresponding author.

## Supplementary Materials

The following supporting information can be downloaded at: https://www.mdpi.com/article/10.3390/microorganisms13020464/s1, Figure S1: Screening of conditions for LPS-induced inflammation model in Caco-2 cells; Figure S2: Cell viability assay.
