# Supplementary material for: Characterization and Anti-Inflammatory Effects of Akkermansia muciniphila-Derived Extracellular Vesicles
Source: Microorganisms. 2025 Feb 19;13(2):464. doi: 10.3390/microorganisms13020464 (PMC11858061; doi:10.3390/microorganisms13020464)
Supplement: Supplementary file 1 [file microorganisms-13-00464-s001.zip › microorganisms-3464011-supplementary.pdf]

Figure S1

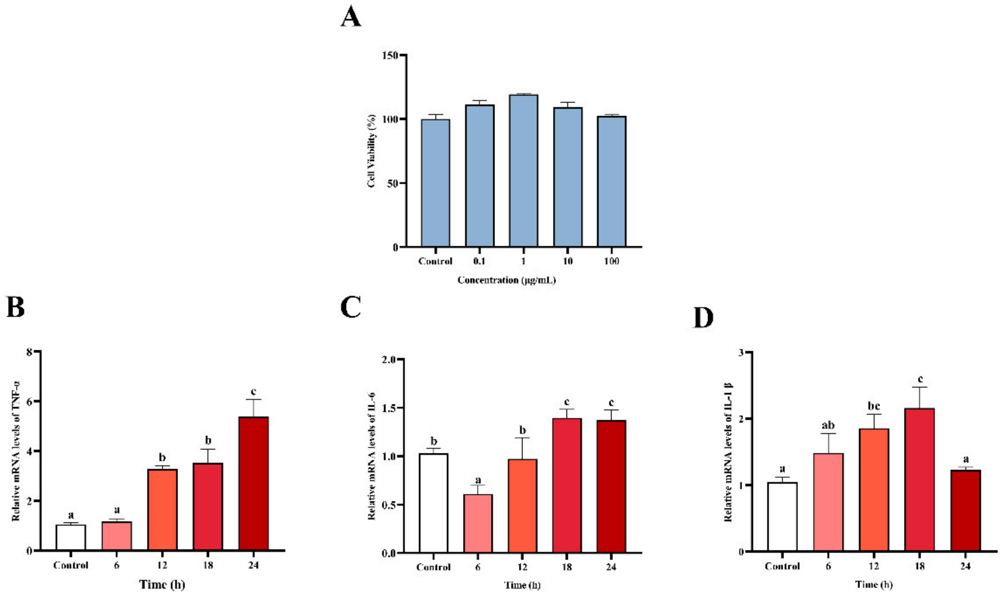

Figure S1. Screening of conditions for LPS-induced inflammation model in Caco-2 cells. A: Effect of different concentrations of LPS on cell viability in Caco-2 cells; B: Effect of LPS on inflammatory cytokines TNF-α mRNA expression; C: Effect of LPS on inflammatory cytokines IL-6 mRNA expression; D: Effect of LPS on inflammatory cytokines IL-6 mRNA expression. Note: Different letters indicate significant differences ($p < 0.05$).

Figure S2

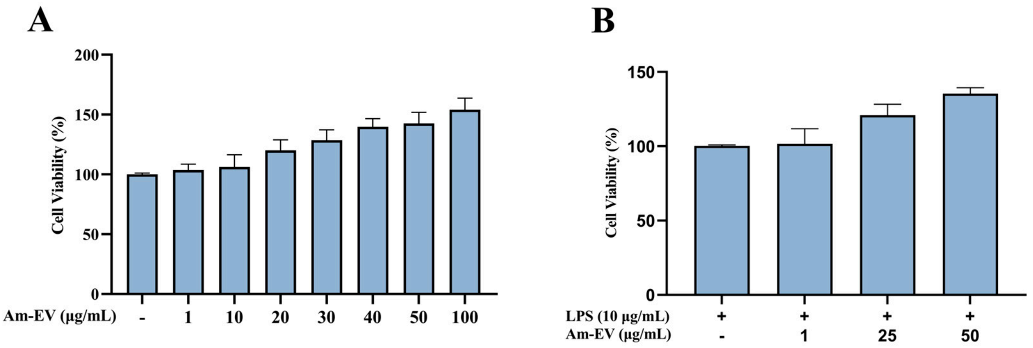

Figure S2. Cell viability assay. A: Effect of different concentrations of Am-EVs on cell viability of Caco-2 cells; B: Effect of different concentrations of Am-EVs on cell viability of Caco-2 cells induced by LPS.
